# Rejuvenation Strategy for Inducing and Enhancing Autoimmune Response to Eliminate Senescent Cells

**DOI:** 10.14336/AD.2024.0579

**Published:** 2025-07-17

**Authors:** Xingyue Wang, Chengyu Zhang, Jiahong Su, Siqi Ren, Xiang Wang, Yinping Zhang, Zijun Yuan, Xinyu He, Xu Wu, Mingxing Li, Fukuan Du, Yu Chen, Shuai Deng, Yueshui Zhao, Xiaodong Wang, Yuhong Sun, Jing Shen, Huijiao Ji, Yunqing Hou, Zhangang Xiao

**Affiliations:** ^1^Laboratory of Molecular Pharmacology, Department of Pharmacology, School of Pharmacy, Southwest Medical University, Luzhou, China.; ^2^Cell Therapy & Cell Drugs of Luzhou Key Laboratory, Luzhou, Sichuan, China.; ^3^South Sichuan Institute of Translational Medicine, Luzhou, Sichuan, China.; ^4^Department of Hepatobiliary Disease, The Affiliated Traditional Chinese Medicine Hospital, Southwest Medical University, Luzhou, Sichuan 646000, China.; ^5^LongmaTan District People's Hospital of Luzhou City, Luzhou 646600, China.; ^6^Department of Pharmacology, School of Pharmacy, Sichuan College of Traditional Chinese Medicine, Mianyang 621000, China.

**Keywords:** senescence, mechanisms of aging, immunotherapy, CAR-T cells, CAR-NK cells

## Abstract

The process of aging, which involves progressive changes in the body over time, is closely associated with the development of age-related diseases. Cellular senescence is a pivotal hallmark and mechanism of the aging process. The accumulation of senescent cells can significantly contribute to the onset of age-related diseases, thereby compromising overall health. Conversely, the elimination of senescent cells enhances the body's regenerative and reparative capacity, thereby retarding the aging process. Here, we present a brief overview of 12 Hallmarks of aging and subsequently emphasize the potential of immune checkpoint blockade, innate immune cell therapy (including T cells, iNKT cells, macrophages, and NK cells), as well as CAR-T cell therapy for inducing and augmenting immune responses aimed at eliminating senescent cells. In addition to CAR-T cells, we also explore the possibility of engineered immune cells such as CAR-NK and CAR-M cells to eliminate senescent cells. In summary, immunotherapy, as an emerging strategy for the treatment of aging, offers new prospects for age-related research.

## 1. Introduction

With the aging of the population, age-related diseases have increased dramatically. Anti-aging research has advanced rapidly to address this situation, including the exploration of the mechanisms of aging. These mechanisms may seem isolated, but the Geroscience Hypothesis suggests that aging mechanisms can progress in concert and may be root-cause contributors to multiple diseases. This suggests that targeting senescent cells tends to weaken other underlying aging mechanisms [1]. Therefore, removing senescent cells to delay the aging process and treat age-related diseases is a highly anticipated approach [2]. However, the body's immune system's ability to eliminate senescent cells decreases with age [3]. Given the effectiveness of immunotherapy in treating tumors, researchers are exploring its application in the field of aging. Currently, new immunotherapies based on CAR technology show broad prospects [4]. However, the primary challenges for this technology are identifying and verifying specific surface targets of senescent cells. In addition, dual-target antagonism and combination immunotherapies have development potential in anti-aging treatment.

In this review, we initially present a brief overview of the mechanisms that characterize the aging process. Highlighting the latest advances in immune-mediated elimination of senescent cells, include immune checkpoint blockade therapy, innate immune cell therapy, and immune cell modification using chimeric antigen technology. We aim to provide a novel and professional perspective for the development of anti-aging therapy.

## 2. Mechanisms of Aging

**Figure 1.**
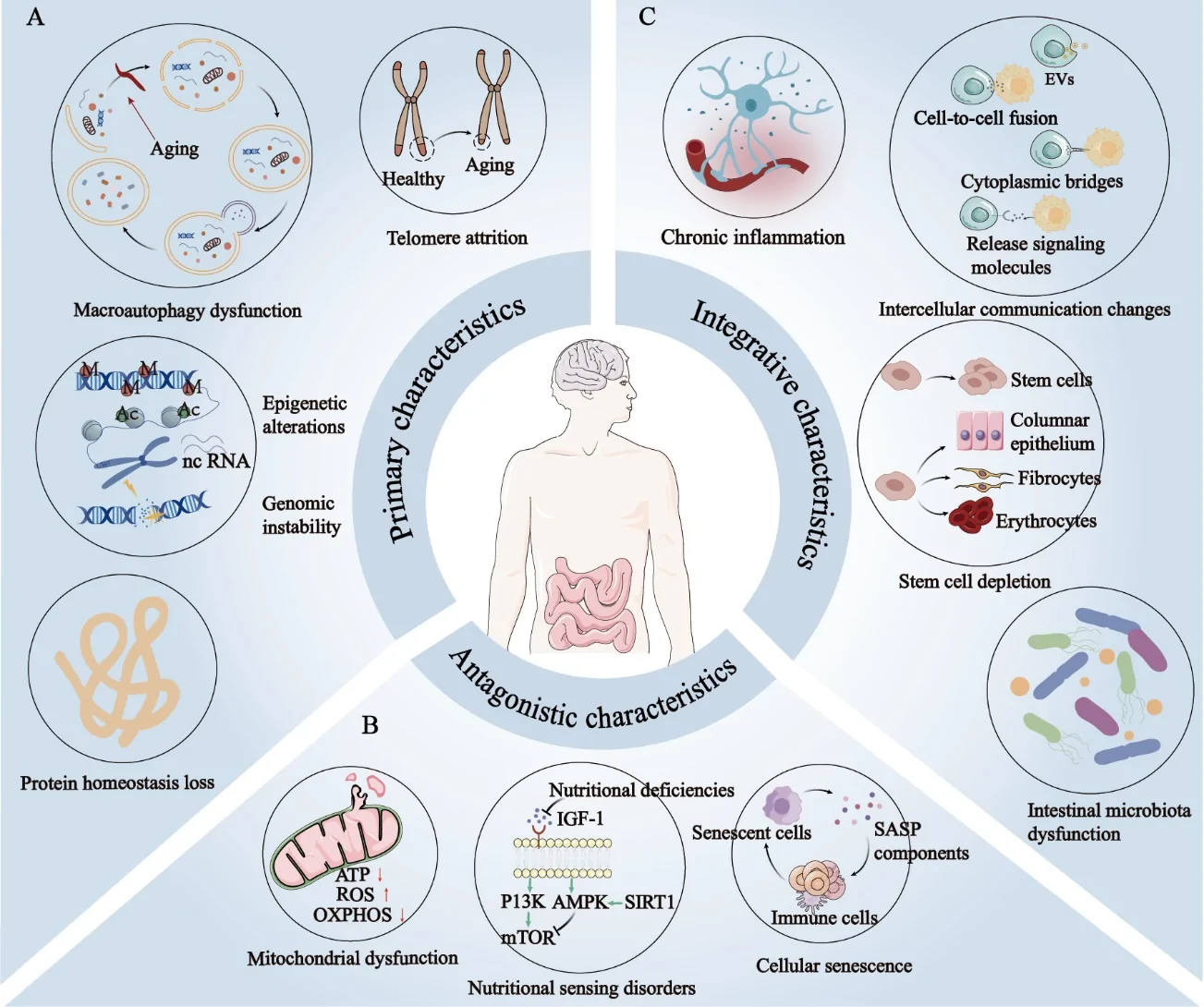
**The mechanisms and features of aging**. The mechanisms of aging can be divided into three categories: (A) primary characteristics (genomic instability, telomere attrition, epigenetic alterations, protein homeostasis loss, macroautophagy dysfunction); (B) antagonistic characteristics (nutritional sensing disorders, mitochondrial dysfunction, cellular senescence); (C) integrative characteristics (stem cell depletion, intercellular communication changes, chronic inflammation, gut microbial dysbiosis). All these will lead to the occurrence of aging. EVs, extracellular vesicles; IGF-1, insulin/insulin-like growth factor 1; P13K, phosphatidylinositol-3-kinase; AMPK, AMPK-activated protein kinase; mTOR, mammalian target of rapamycin; SIRT1, sirtuin1; ATP, adenosine triphosphate; ROS, reactive oxygen species; OXPHOS, oxidative phosphorylatio; SASP, senescence-associated secretory phenotype.

A multitude of factors influence aging. A comprehensive investigation into its characteristics and underlying mechanisms holds the potential to yield novel insights for disease diagnosis and treatment. The characteristics of aging at this stage can be categorized into three domains: primary characteristics (genomic instability, telomere attrition, epigenetic alterations, protein homeostasis loss, macroautophagy dysfunction); antagonistic characteristics (nutritional sensing disorders, mitochondrial dysfunction, cellular senescence); and integrative characteristics (stem cell depletion, intercellular communication changes, chronic inflammation, gut microbial dysbiosis). Figures 1A, 1B, and 1C summarize the mechanisms of aging (Fig. 1).

### 2.1 Primary characteristics

The primary features of aging are the initiating factors that induce damage. These features cause damage at the cellular level and lead to detrimental consequences.

Genomic instability is considered the main characteristic of aging. The field of genomics is responsible for the storage of genetic information. The proper functioning of organisms relies heavily on the precise regulation of their genome maintenance system [5]. Influenced by both exogenous and endogenous factors, the genome can undergo a range of alterations, including gene mutations, deletions, translocations, telomere shortening, single-strand or double-strand breaks (SSB or DSB) [6], chromosomal rearrangements, nuclear structural defects, as well as integration of viruses or transposons and these are key contributors to genomic instability [7]. Fortunately, the organism can initiate additional repair processes to rectify the imbalanced physiological system, ensuring that nearly all of the 10^4^-10^5^ genes undergoing mutations daily in each nuclear genome are effectively repaired. Common mechanisms include processes such as base-excision repair (BER), microhomology-mediated end joining, single-strand annealing, nonhomologous end-joining, and homologous recombination [8, 9]. For example, BER capacity tends to decline with age in mammals. The compromised DNA repair capacity results in heightened genomic instability, which may be one of the reasons for the increased degree of DNA damage and mutation during aging [10]. Previous studies have demonstrated that Sirtuin 6 in centenarians significantly enhances the stimulation of DNA DSB repair and improves genome maintenance [11]. The aging process is accompanied by an increase in endogenous factors, resulting in a decline in DNA repair capacity and the accumulation of damage [6]. This leads to an elevation in genomic instability. When additional repair procedures fail to adequately compensate, the accumulation of genetic damage occurs throughout an individual's lifespan, triggering the biological mechanisms associated with aging [12]. Similarly, DNA damage impairs stem cell functionality, impedes tissue regeneration, and enhances susceptibility to aging and diseases [13]. So far, there is no conclusive evidence that normal mutation rates are the cause of aging [14]. On the contrary, the absence or impairment of DNA repair mechanisms has been implicated in the process of aging, as supported by numerous research findings. However, the current lack of evidence that the further delay of aging and reduction in age-related diseases, through the mitigation of nuclear DNA mutation burden or enhancement of repair mechanisms.

The issue of telomere damage, a prominent sign of aging, has gained significant attention in professional circles. The G-rich tandem repeat sequence (TTAGGG)_n_ on human chromosomes [15, 16], known as telomeres, plays a crucial role in maintaining genomic stability [17]. The length of telomeres is considered to be the biological clock, reflecting the age and health status of cells. In somatic cells, the inactivation of telomerase results in a gradual reduction of telomere length. Dysfunctional telomeres induce DNA damage responses, provoke the expression of proinflammatory factors, and halt cell cycle progression, ultimately leading to cellular senescence [18]. Many studies have consistently shown an inverse relationship between telomere length and age [19, 20]. Therefore, the potential of delaying aging through telomere length extension is currently being investigated by scientists, with a particular focus on the role of telomerase. The shelterin complex in humans binds to telomeres, facilitating the formation of T rings and preventing the activation of DNA damage responses [21]. Therefore, a deficiency in shelterin can result in telomere decapitation or even telomere collapse. Whether caused by telomere shortening, damage to telomeric DNA, or depletion of telomeric proteins, telomere damage can be used to characterize the trajectory of aging [22].

The term "epigenetic regulation" refers to the heritable changes in phenotype or gene expression without alterations in the DNA sequence [23, 24]. Epigenetic changes include modifications in DNA methylation patterns, aberrant histone modifications, loss of heterochromatin, reorganized three-dimensional (3D) genomic structures, and dysregulated RNA modifications [22]. These alterations can impact the regulation of genes, resulting in a range of age-related ailments. Studies have shown that disruption of epigenetic information leads to signs of aging in mice, which can be effectively reversed by restoring the integrity of the epigenome [25]. The DNA methylation levels of certain genes exhibit a positive correlation with age. This methylation has an inhibitory effect on gene expression, affecting cell function and accelerating the aging process. The methylation patterns of specific sites, referred to as "epigenetic clocks," are closely associated with biological age and serve as indicators of aging [26]. In summary, these mechanisms impact the local accessibility of genetic material and aberrant transcription, thereby contributing to the progression of aging and age-related diseases.

Aging and related diseases are associated with imbalances in protein homeostasis. When the capacity of the protein homeostasis network and the integrity of the proteome are challenged, proteins undergo misfolding and aggregation. This frequently serves as the primary determinant of susceptibility to neurological disorders in elderly individuals and assumes a pivotal role in cellular senescence [27]. The cells can maintain protein homeostasis through various quality control mechanisms [28]. The decline in protein homeostasis with age increases the risk of abnormal protein aggregate accumulation [29]. Common mechanisms for protein stabilization encompass: (1) The role of ribosomes enhances the accurate translation of RNA into proteins, ensuring protein stability [30]. (2) Lysosomes selectively degrade proteins containing the KEFRQ motif through chaperone-mediated autophagy (CMA) [31]. (3) The autophagosomes can sequester non-protein structures and undergo degradation through fusion with lysosomes, facilitating the process of autophagy and eliminating protein aggregates within cells [32]. Imbalances in the core components that maintain protein homeostasis (the molecular chaperone status, autophagy-lysosomal system, ubiquitin-proteasome system [33, 34], and stress response pathway [35]) have also been implicated as hallmarks of aging and various age-related diseases [7]. Targeting the loss of proteostasis plays a crucial role in regulating healthy aging or extending life span [36].

Autophagy is a cellular self-degradation mechanism that involves lysosomes to break down damaged proteins, organelles, or pathogens for re-use by the cell [37]. The core regulatory mechanism of autophagy controls the process through inhibition of the mechanistic target of rapamycin (mTOR) or activation of 5′ AMP-activated protein kinase (AMPK) [38]. It is essential for upholding protein homeostasis, ensuring metabolite availability, defending against pathogens, and regulating aging [39]. The expression of autophagy-related genes is closely associated with longevity. Researchers found that the long lifespan of C. elegans was associated with autophagy-related genes, including Bec-1, Lgg-1, Atg-7, and Atg-12 [40]. Additionally, during normal brain aging, the expression of Atg-5, Atg-7, and Bec-1 decreased [41]. Stimulating autophagy tends to promote longevity. Spermine as an autophagy inducer, inhibits autophagy EP300, an endogenous inhibitory factor to delay the progression of some age-related diseases [42]. Autophagy can protect the adaptive cells from chronic inflammation in the bone marrow niche of aging mice and improve the regenerative potential of old hematopoietic stem cells. Targeting autophagy can improve the elderly with chronic inflammatory diseases and systemic tissue degeneration [43]. It is worth noting that autophagy-related genes can also be upregulated during aging; some conditions that inhibit autophagy can slow the aging phenotype [44]. Further research on the mechanisms of autophagy regulation will help to develop therapeutic strategies for aging.

### 2.2 Antagonistic characteristics

The body's response to injury manifests as antagonistic properties, which in the context of aging involve dysregulation of the nutrient-sensing network, cellular senescence, and impaired mitochondrial function.

The nutritional sensing network is highly conserved in the evolutionary process. It enables organisms to perceive changes in external nutrients and make appropriate responses, which are necessary for the survival of organisms [45]. The nutrition-sensing network consists of extracellular ligands, receptor tyrosine kinases, and associated cell signaling cascades [7]. The central nutrient-sensing metabolic pathways include the insulin and insulin-like growth factor pathway, the mTOR, AMPK, and the Sirtuins protein pathway [46]. The nutrition-sensing network can modulate cellular activity levels in response to variations in nutrient and stress states. This modulation involves either promoting or inhibiting anabolic processes [45, 47]. Calorie restriction in many animal species extends life span and may slow the aging process [48]. The growth hormone axis, as the first growth axis, consists of interconnected hormones and receptors located on the hypothalamus-pituitary-target organs, thereby playing a pivotal role in perceiving nutritional cues and cellular energy status [7]. Innate defects cause dwarfism, and youth suppression is beneficial to the body. Additionally, signaling pathways that rely on the tyrosine kinase provide additional targets for interventions on metabolic aging [7]. As pointed out by Weaver, the nutritional sensing network of fruit flies can be regulated by starvation-induced neuronal tissue modification. This modulation has the potential to extend lifespan and confer beneficial effects on aging [49].

The process of cellular senescence is a stress response that involves a series of functional and morphological changes during the arrest of the cell cycle [50]. It is characterized by the generation of complex secretion (SASP) and long-term cell cycle arrest and is often irreversible [51]. Cellular senescence serves as a pivotal factor in aging or age-related diseases. The SASP is a complex collection of inflammatory factors, chemotactic factors, and growth factors secreted by senescent cells [2, 52]. It triggers secondary aging processes, disrupts tissue homeostasis, and ultimately results in the loss of damage to tissue repair and regeneration [53]. Cellular senescence coordinates tissue remodeling through three sequential processes, including recruiting immune cells SASP [54]. However, immunosenescence, a decline in immune function during aging, can result in chronic low-grade inflammation that adversely affects health [55]. Analysis of proteomics and transcriptomics datasets has shown that senescent cells upregulate one or more senescent cell anti-apoptotic pathways (SCAPs). Consequently, initial research efforts have primarily focused on devising strategies targeting these anti-apoptotic pathways. Currently, several targeted drugs have entered clinical trials [1].

The mitochondria function as vital energy suppliers and their dysfunction can lead to severe damage to cellular energy conversion [56]. Unfortunately, mitochondrial function gradually becomes abnormal during aging. Mitochondrial respiratory chain complexes I and III generate reactive oxygen species (ROS), which can lead to oxidative damage and mutations in mtDNA. Mutations in damaged mtDNA further affect the respiratory chain and generate more ROS, resulting in a vicious cycle of ROS and the accumulation of mtDNA mutations. ROS and mtDNA mutations are among the mitochondrial aging biomarkers. They affect mitochondrial function and accelerate the aging process [22]. In addition, mitochondrial dynamics also play an important role in the aging process, and promoting the mitochondria of dynamic balance and plasticity is helpful to healthy aging [57]. Apoptosis and senescence are regulated by similar mitochondria-dependent mechanisms. At the same time, inhibiting the inflammation induced by a few mitochondrial outer membrane permeability (miMOMP) could be a therapeutic avenue for age-related diseases [58].

### 2.3 Integrative characteristics

The integrative characteristics emerge when the damage caused by the primary and antagonistic traits cannot be compensated for, directly impacting tissue homeostasis and function.

The distinguishing features of stem cells include their capacity for self-renewal and multi-differentiation. They play a crucial role in the repair and regeneration of adult stem cells within the human body. Stem cell therapy has been successfully employed in various medical conditions, including age-related diseases [18, 59, 60]. The study has found that using small extracellular vesicles (sEVs) from mesenchymal stromal/stem cells (MSCs) can reduce SASP and help rejuvenate senescent endothelial cells by increasing miR-146a-5P [61]. According to reports, stem cell regeneration can be induced through a process called "cell reprogramming", which converts adult stem cells into induced pluripotent stem cells (iPSCs). This transformation reduces the aging marker p16 and extends telomeres, thereby endowing these cells with the ability to repair aging tissues [62]. Small molecular-based chemical reprogramming can control human cell reprogramming in a simple way, with great potential for medical regeneration [63]

Aging is associated with changes in intercellular communication (SASP, juxtaglsecretory signal, intercellular fusion, cytoplasmic bridge, etc.). This leads to impaired homeostasis and hormonal regulation, including defects in insulin-IGF1 signaling and sex hormones [7]. The classical SASP comprises soluble factors, growth factors, and extracellular matrix (ECM)-remodeling enzymes, whereas the new SASP encompasses extracellular vesicles (EVs) as well as noncellular metabolites and ions [64]. The process of aging results from the integration of multiple signaling pathways, focusing on two major pathways: the p53 pathway and the pRb pathway [65]. The former mediates aging resulting from DNA damage and telomere dysfunction. But the latter mediates aging caused by chromatin degradation, oncogenes, and various stress responses [66-68]. The latest research suggests that tumor necrosis factor-α (TNF-α) signaling, including activation of the nuclear factor kappa-B (NF-κB) pathway, mitogen-activated protein kinase (MAPK) pathway, and phosphatidyl-inositol 3-kinase/serine-threonine kinase (PI3K-AKT) pathway, controls the cellular senescence phenotype by regulating SASP production and inducing cell cycle arrest [22].

Chronic inflammation is a major feature of aging and can induce senescence through the activation of the GH/IGF1/PI3K/AKT/mTORC1 pathway [7]. Inflammatory aging is a pivotal factor contributing to the process of aging and deterioration in immune function. TORC1-S6K-Syx13 signaling pathway can regulate hepatitis, reduce immune senescence, and prolong lifespan through the lysosomal system [69]. The release of SASP from senescent cells can cause chronic inflammation and induce normal cell senescence. This process accelerates immune senescence and eventually leads to the worsening of the inflammatory and senescence cycle [70]. The study of people aged 45 to 115 found that long-lived people had strong anti-inflammatory abilities. Therefore, it is proposed that inflammation is one of the driving factors of aging, and drug research in this aspect has great potential for slowing down aging [71]. Several studies have shown that inflammation can be improved, and aging can be intervened by improving diet, promoting immune regeneration, clearing senescent cells, and anti-inflammatory drugs [72-74]. In summary, chronic inflammation plays an important role in aging, and its regulation will open new avenues for anti-aging drug research.

The composition of the intestinal microbiota maintains a diverse microbial population in long-lived individuals. Older adults with poor health exhibit reduced bacterial diversity and an increased proportion of pathogenic bacteria [75, 76]. So far, findings have indicated that alterations in the gut microbiome and metabolic dysregulation in the host can contribute to age-related diseases [77-79]. Further evaluation of the role of remodeling gut microbiota in rescuing bile acids homeostasis and mitigating age-related diseases was conducted by co-feeding female, male, young, and old mice [80]. Researchers have found that interventions based on exercise and diet can reshape microbiota composition [81]. The administration of probiotics enhances muscle condition in rodents [82]. The transplantation of fecal microbiota from young to old mice effectively reversed aging characteristics in older mice [83]. The relationship between intestinal microorganisms and cell aging is bidirectional. Metabolites produced by intestinal microorganisms have the potential to decelerate cellular senescence, while cellular senescence can lead to intestinal cell dysfunction and dysbacteriosis [84]. Hence, targeting the intestinal microbiota through intervention holds potential as an approach for treating aging.

## 3. Immunotherapy for the elimination of senescent cells

Currently, targeting strategies for aging treatment can be broadly categorized into three main categories: the selective elimination of senescent cells (senolysis), immune-based clearance of senescent cells, and SASP neutralization technology [85]. Senolytics, an emerging category of anti-aging drugs, target the signaling pathways of senescent cells, disrupting their anti-apoptotic mechanisms and enabling selective elimination of these cells [1, 86]. Previously, the majority of anti-aging research has focused on the development of small molecule therapies. However, these therapies primarily target molecular dependencies that have not been clearly delineated in senescent cells, necessitate prolonged and repeated administration. Secondly, the population of senescent cells targeted by small molecule therapies is also uncertain [87, 88]. Subsequent studies have shown that there are many common biological processes related to cancer and aging [89]. Immunotherapy is an important means of cancer treatment. Researchers have introduced immunotherapy into the exploration of aging in the path of deepening aging research (Table 1). The induction and augmentation of immune response can facilitate the elimination of senescent cells. It also alleviates chronic inflammation, enhances overall health status, and mitigates susceptibility to chronic diseases. We will commence with conventional approaches such as immune checkpoint blockade, innate immune cell therapy, and chimeric antigen receptor immune cell therapy. The main discussion will focus on their potential in aging, thereby laying a foundation for subsequent research on aging.

**Table 1:** Immune clearance of senescent cells.

Interventions	Target Cells	Results	Ref
**NKG2D CAR-T cells**	Fibroblasts	Identify and eradicate cells expressing NKG2DLs	[145]
**uPAR CAR-T cells**	Liver fibrosis cells	Reverse liver fibrosis	[143]
	Lung adenocarcinoma cells	Improvement to metabolic dysfunction	[178]
**Anti-PD-1 antibody**	p16^+^ cells	Reduces the total number of p16^+^ cells in vivo	[90]
**Senolytic vaccine**	Gpnmb-positive cells	Improved normal and pathological phenotypes associated with aging and extended the male lifespan of progeroid mice	[179]
**CD153-CpG vaccine**	Senescent T cells	Reduced the number of senescent T cells	[180]
**NK cells**	Senescent CD4^+^ T cells	SASP-related factors decreased (IL-6, IL-8, IL- 1α, IL-17, MIP-1α, MIP-1β, and MMP1) and Senescent T cells decline	[181]
**iNKT cells**	Senescent cells in bleomycin-induced lung injury model	Reverse adverse metabolic phenotypes in the HFD mouse model and the fibrosis induced by lung injury	[116]
**CD4^+^ T cells**	Fibroblasts	Recognition of the HCMV-gB antigen to eliminated senescent cells	[112]
**Macrophages**	β cells	The clearance of senescent β cells and reduced terminal/late senescence in UPR-deficient NOD mice.	[168]

Gpnmb, glycoprotein nonmetastatic melanoma protein B; HFD, high-fat diet; HCMV-gB, human cytomegalovirus glycoprotein B; UPR, unfolded protein response; NOD, non-obese diabetic.

### 3.1 Immune checkpoint blockade

Immune checkpoint blockade therapy is one of the hottest immunotherapies nowadays. It broadens the landscape of cancer treatment and instills new hope for non-cancerous diseases. Programmed cell death protein 1 (PD-1)/programmed cell death-ligand 1 (PD-L1) immune blockade therapy stands out as a prototypical example. The expression of PD-L1 in senescent cells is heterogeneous, and with age, there is a gradual accumulation of PD-L1^+^ senescent cells in the body [90, 91]. Therefore, utilizing PD-1/PD-L1 immune checkpoint blockade therapy to eliminate senescent cells expressing PD-L1 shows promise as an anti-aging treatment strategy [90].

A clinical study reported on 14 patients with non-small cell lung cancer who received treatment with anti-PD-1 inhibitors (nivolumab and pembrolizumab)/anti-PD-L1 inhibitor (atezolizumab). Hair repigmentation (HR) occurred in 13 patients, resulting in a conversion of white hair color to black [92]. In a subsequent analysis of the report, Manson pointed out that hair repigmentation following PD-1/PD-L1 inhibitor therapy is not limited to lung cancer patients. He described a case of Hodgkin lymphoma in which, one month after treatment with nivolumab, the patient reported systemic HR phenomena [93]. This phenomenon is commonly regarded as an adverse reaction in cancer treatment, but it may indicate that PD-1 is expected to be a potential target for anti-aging therapy. In the study of Alzheimer's disease (AD), researchers observed a significant increase in the expression levels of PD-1 and PD-L1 in patients with AD. PD-1 influences the hyperphosphorylation of Tau protein by regulating the activity of glycogen synthase kinase 3β (GSK-3β), a crucial pathological characteristic of AD. Blockade of the PD-1/PD-L1 axis significantly reduced GSK-3β activity and Tau protein hyperphosphorylation in the AD mice model. This had a positive effect on cognitive function in AD mice [94]. Subsequently, several scholars have investigated the potential of Nucleotide-binding oligomerization domain-containing protein 2 receptor agonists in conjunction with a PD-L1 inhibitor for the treatment of AD. This offers novel insights to enhance patient prognosis and quality of life [95].

Under normal physiological conditions, the interaction between PD-1 and its ligand PD-L1 plays a pivotal role in modulating the immune system's response to infectious pathogens, neoplastic cells, and autoimmune diseases [96]. In certain disease states, such as cancer, tumor cells can evade the immune system by upregulating PD-L1 expression, enabling them to avoid recognition and elimination by immune cells. This compromises the immune system's effective control over tumor cells. Similarly, immune checkpoints also exert a negative regulatory function in the immunosurveillance of senescent cells. The study has shown that PD-L1 expression is significantly elevated in p16^+^ cells from older mice, exceeding the levels in younger mice. Despite being in the state of SASP, these senescent cells expressing PD-L1 can effectively evade immune surveillance and attack, achieving immune escape. PD-L1 expression is associated with high levels of SASP [90]. Additionally, immune cell senescence in type 1 diabetes is also linked to the expression of PD-1 [97]. It is worth noting that the upregulation of PD-L1 in aging and aged tissues is regulated by multiple mechanisms. For instance, the SASP triggers activation of the JAK-STAT signaling pathway through the release of a range of cytokines, including interferon and other proinflammatory cytokines, further promoting an increase in PD-L1 transcription levels. In addition, the transcription level of PD-L1 is regulated by the activation of transcription factors, including STAT1, STAT3, NF-kB, and Myc [91]. The study conducted by Nakanishi et al. revealed that the upregulation of PD-L1 heterogeneity in senescent cells is partially attributed to the enhanced transcriptional activity mediated by e2f1 and the reduced proteasome activity [90]. PD-L1 expression is also stabilized by the p16-mediated CDK4/6 inhibitory pathway [98]. The administration of the anti-aging drug rapamycin can effectively inhibit the SASP process in senescent cells, reducing the expression of PD-L1. In mouse models, this intervention has demonstrated a remarkable capacity to extend lifespan [91].

In fact, in mouse models of natural aging or nonalcoholic steatohepatitis, PD-1 antibody treatment appears to reduce the number of p16^+^ cells through a CD8^+^ T cell-dependent mechanism [90]. Programmed cell death ligand 2 (PD-L2) can be upregulated in various types of senescent cancer cells. However, the absence of PD-L2 in the tumor microenvironment leads to CD8^+^ T cell-mediated attack on senescent cells while preventing the recruitment of myeloid-derived suppressor cancer cells, thereby enhancing the susceptibility of tumors to immune surveillance [99]. Further research is required to ascertain the involvement of PD-L2 in the accumulation of senescent cells and the manifestation of age-related diseases.

### 3.2 Innate immune cell therapy

**Figure 2.**
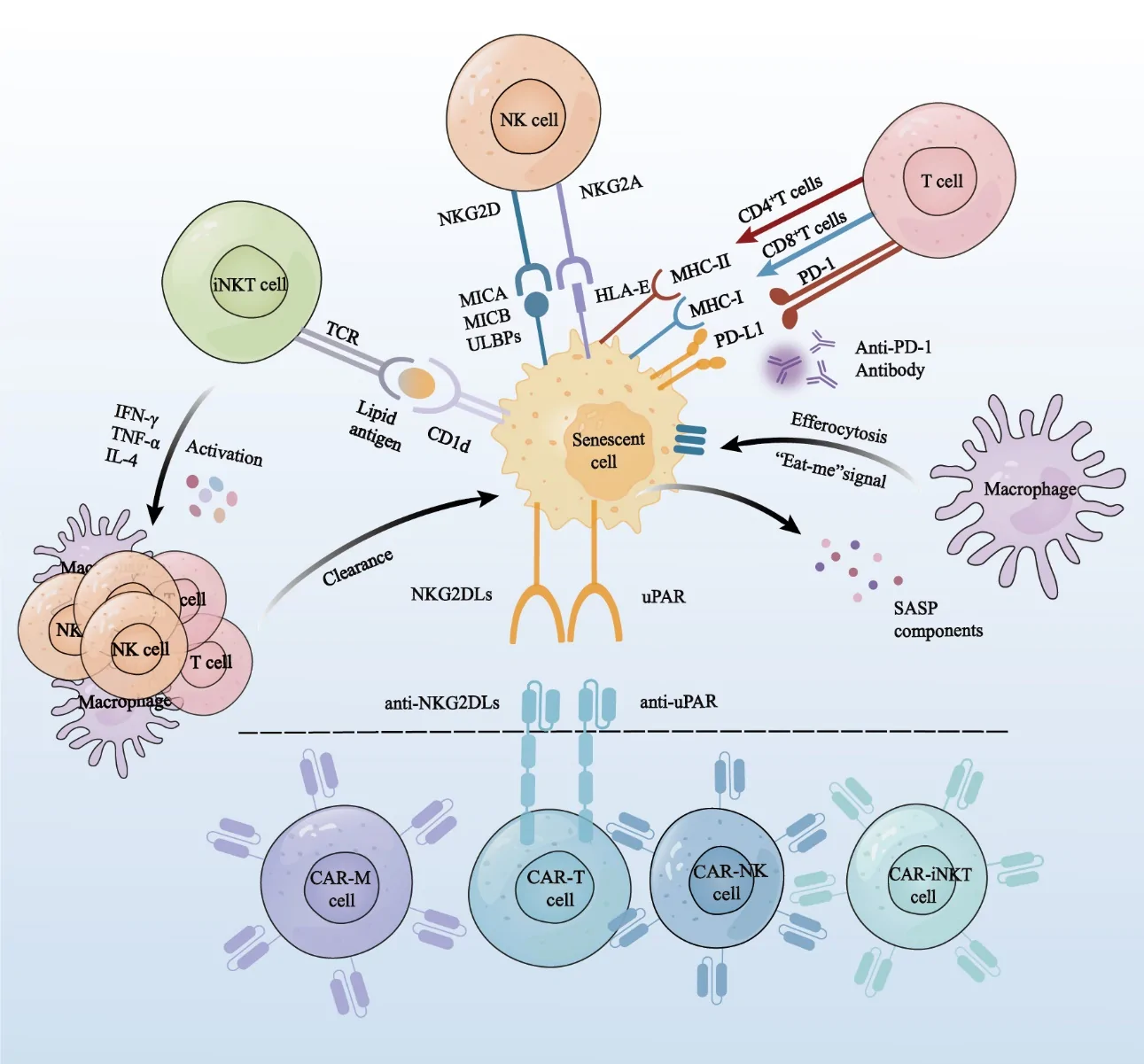
**Distinct immune cell types or immune activation mechanisms are involved in the elimination of senescent cells**. The immune cells recognize and bind specific ligands on senescent cells, such as PD-L1, MHC-I, MHCII, and NKG2DLs, in order to eliminate the senescent cells.

Aging phenotype expression depends on the environment and triggers. Apart from the DNA damage response, cellular senescence can also be induced by various stressors, including but not limited to oncogenes, genomic instability, mitochondrial dysfunction, reactive metabolites, inflammation, inactivation of specific tumor suppressor genes, epigenetics, and treatment-induced stress [53, 100]. The clearance of senescent cells in various tissues requires the involvement of distinct immune cell subsets (Fig. 2). Consequently, it is necessary to investigate the specific surface targets of senescent cells in diverse diseases.

### 3.2.1 NK cells

Natural killer (NK) cells constitute the fundamental component of innate immune cells and possess the ability to exert direct effector functions without requiring prior activation. Sansoni et al. discovered that NK cells possess not only potent anti-cancer effects but also exhibit remarkable efficacy in combating aging processes. The activity of NK cells is significantly higher in centenarians compared to middle-aged individuals. It is hypothesized that maintaining high NK cell activity is conducive to longevity [101].

NK cells comprise various subpopulations, including NKG2A, NKG2C, NKG2D, KIR, and NCRs (NKp30, NKp44, and NKp46). They possess an inherent capacity to recognize and eliminate malignant cells as well as virus-infected cells [102, 103]. Additionally, NK cells can exert their effects by activating the interaction between their own receptors and ligands on senescent cells, thereby releasing cytotoxic particles such as granzyme and perforin [104]. For example, NKG2D-activated receptors bind to target cell surface ligands MICA and MICB to activate NK cytotoxicity. Another important receptor, NKG2A, interacts with the major histocompatibility complex (MHC) class I molecule human leukocyte antigen E (HLA-E) to suppress NK cytotoxicity and maintain immune homeostasis [105, 106]. Multiple chemotherapeutic agents, such as Bortezomib, Melphalan, and Doxorubicin, have been shown to upregulate DNAM-1 and NKG2D ligands to enhance the lysis of multiple myeloma cells by NK cells [107, 108]. In addition, NK cells exhibit a diverse range of activities. The SASP enhances the initial stage of NK cell-mediated immune response, however, excessive accumulation of senescent cells can lead to SASP-induced inhibition of immune response [109]. Increasing the clearance of senescent cells by NK cells facilitates a positive immune response. With the development of research, NK cells will reach a new level in the treatment of aging-related diseases.

### 3.2.2 T cells

The total T cell pool undergoes significant changes with age, characterized by a decline in the naive T cell population and an expansion of the memory T cell population. Meanwhile, the diversity of the T cell receptor (TCR) is concurrently attenuated, impacting the onset and progression of age-related ailments [110]. The role of T cells in immune surveillance and healthy longevity is pivotal. CD4^+^ T cells regulate immune responses by producing various cytokines, allowing them to recognize MHC II molecules on tumors or senescent cells and directly eliminate senescent tumor cells [111]. For instance, CD4^+^ T cells can eliminate senescent hepatocytes before liver cancer develops in mice and inhibit mouse liver cancer [111]. Additionally, CD4 CTLs can eliminate senescent fibroblasts in the skin of elderly individuals by targeting the glycoprotein B antigen (HCMV-gB) derived from human cytomegalovirus, using an HLA-II dependent mechanism [112]. The increased expression of MHC-I and the molecular mechanisms required for antigen processing and presentation in senescent cells make them more easily recognized and killed by CD8 T cells [113]. Emerging therapies that aim to deplete pathogenic T cells or enhance protective T cell responses by resetting the immune system tolerance are being widely employed as a strategic approach [110]. Additionally, regulating the crosstalk between T cells and the microbiota can effectively preserve intestinal integrity, and prevent bacterial translocation and related inflammation, ultimately delaying age-related diseases [114].

### 3.2.3 iNKT cells

Invariant natural killer T (iNKT) cells are regarded as a crucial connection between the innate and adaptive immune systems [115]. The study by Arora et al. revealed that iNKT cells enhance the removal of senescent cells through cytotoxin secretion and regulation of other immune cell activity [116]. The iNKT cell population in humans and mice exhibits distinct expression patterns of TCR chains, leading to their rearrangement and subsequent formation of diverse pairing chains. The specific TCR confers iNKT cells with a distinctive capacity to recognize both endogenous and exogenous lipid antigens presented by the MHC-I like molecule CD1d, particularly glycolipid antigens [117], such as α-galactosyl ceramide (α-GalCer) [118]. The activated iNKT cells secrete cytokines, including interferon-γ (IFN-γ), TNF-α, and IL-4. These cytokines stimulate other immune cells [119], such as macrophages, B cells, and CD8^+^ T cells [120], to combat infections, autoimmune diseases, and tumorigenesis. Activation of iNKT cells with αGalCer leads to a reduction in senescent preadipocytes in chronic high-fat diet (HFD)-fed mice. Similarly, senescent cells were reduced in lung-injured mice, improving their survival [116]. Activating iNKT cells can be used to treat a variety of chronic diseases, and further provides a theoretical basis for anti-aging research. Significantly, the presence of iNKT cells in peripheral blood is limited, accounting for approximately 0.001% to 1% [121]. Therefore, culturing allogeneic iNKT cells on a large scale for cellular therapy to counteract the aging process poses significant challenges. Utilizing hematopoietic stem cell (HSC) genetic engineering technology and *in vitro* differentiation techniques, researchers successfully generated high-quality and high-purity human allogeneic HSC-engineered iNKT cells (^Allo^HSC-iNKT) [122]. The increasingly optimized techniques propel iNKT therapy as a promising advancement, offering the potential to overcome its application limitations.

### 3.2.4 Macrophages

Macrophages are essential components of the host's innate immune system for defense against pathogens and cancer [123]. They possess the ability to differentiate into two distinct subgroups: pro-inflammatory (M1) and anti-inflammatory (M2) phenotypes. Research has revealed that the decreased expression of epidermal IL-34 in aging skin may lead to an increased M1/M2 ratio in the dermis, consequently resulting in inflammatory aging [124]. The interaction between senescent cells and immune cells can impact the functionality of the immune system. Senescent cells recruit immune cells through SASP, leading to the induction of senescence and dysfunction in these immune cells, ultimately resulting in the continuous and excessive accumulation of senescent cells [3]. On the other hand, senescent cells secrete a variety of chemokines that attract immune cells and initiate local inflammation [125], while simultaneously facilitating macrophages in their clearance [126]. For example, macrophages clear senescent cells from the mouse uterus and maintain postpartum uterus function in mice [127]. Similarly, macrophages can remove pre-malignant senescent hepatocytes involved in tumor immune surveillance [111]. Macrophage therapy for aging is still in its nascent stages, necessitating further studies to validate its efficacy and safety.

## 3.3 Chimeric antigen receptor immunocytotherapy

### 3.3.1 CAR-T cells

#### 3.3.1.1 Principles and Optimization Strategies

The concept of CAR-T cell therapy is based on the principle of TCR, utilizing the functionality of T cells and antibody specificity to precisely eliminate diseased cells [4, 128]. The key to CAR-T cell therapy lies in the precise design of CAR molecules. CAR is an artificially engineered receptor molecule, consisting of antigen-binding extracellular domains (single-chain variable fragment (scFv) and hinge region), transmembrane domains, and intracellular domains (costimulatory domains and signal transduction domains) [129]. The effectiveness and safety of CAR-T cell therapy rely on the specificity and affinity of scFv towards the antigen. Other crucial factors include the impact of the hinge region's structure on CAR molecule expression and function, provision of a second activation signal through the costimulatory domain, and initiation of T cell activation signal via configuration of CD3ζ chain and immunoreceptor tyrosine-based activation motif [130-133].

**Table 2:** Surface markers of senescent cells.

Surface Markers	Cell Type	Aging Model/Detection Pathway	Ref
**B2M**	Bladder cancer cells	Senescence was induced by the chemotherapeutic drug doxorubicin	[182]
**NOTCH1**	Fibroblasts	Senescence induction by oncogenic MEK, DNA damage, or RIS was confirmed by flow cytometry	[183]
**PD-L1**	Fibroblasts	Senescent cells were induced by Nutlin-3a and DNA damage	[90]
**GPNMB**	Vascular endothelial cells	Senescence was induced by feeding a high-fat diet (HFD)	[179]
**uPAR**	Liver fibrosis cells and lung adenocarcinoma cells	Replication induction, MEK and CDK4/6 inhibitors induced senescence, and CCl_4_ induced liver fibrosis	[143]
**MICA, MICB, ULBP2**	Lung fibroblast cell	SA-β-Gal staining was used to detect the senescence marker p16^INK4a^ mRNA expression; Replication depletion, DNA damage, and triggering oncogenes induce senescence	[145]
**CD38**	Alveolar epithelial cells	The intratracheal (i.t.) bleomycin-induced lung fibrosis mouse model	[184]
**DEP1, NTAL, EBP50, STX4, VAMP3, ARMX3, B2MG, LANCL1, VPS26A and PLD3**	Eladder cancer cell	Proteomic analysis was first performed, followed by Western blot of senescence-specific targets, sucrose gradient fractionation of senescent EJp16 whole cell lysates, and immunofluorescence verification	[185]
**CD44**	Vascular endothelium	Serial passages were used to induce replicative senescence in vitro	[186]
**DPP4(CD26)**	Fibroblasts	The identification of DPP4 was achieved through the utilization of MS analysis	[187]
**Oxidized vimentin**	Fibroblasts	Senescence was induced by treatment with bleomycin	[188]
**DcR2**	Renal tubular epithelial cell	Renal fibrosis was induced by streptozotocin in mice with diabetic nephropathy	[189]
**CD47**	Fibroblasts, Liver stellate cells, and Epithelial cells	Cell senescence was induced using CDK4/6 inhibitors, DNA damaging agents, gamma irradiation, or serial passaging	[190, 191]

MEK, mitogen-activated extracellular signal-regulated kinase; RIS, HRAS^G12V^-induced senescent; HFD, high-fat diet; SA-β-Gal, β-galactosidase; DPP4, dipeptidyl peptidase 4; MS, mass spectrometry.

Currently, CAR-T cells have undergone five rounds of upgrades and optimizations. Among them, the second generation targets CD19 and incorporates the 4-1BB costimulatory domain resulting in the world's first marketed CAR-T cell product, CTL-019 therapy [134, 135]. The fifth-generation CARs have incorporated the IL-2Rβ cytokine receptor and STAT3 binding domains, building upon the foundation of the second generation. This enhances TCR and JAK-STAT signaling pathways, ultimately augmenting the proliferation and activation potential of CAR-T cells [136]. The preparation of CAR-T cells typically involves utilizing autologous T cells from patients. Although this method can avoid triggering immune rejection, its high cost cannot be ignored [137]. The activated CAR gene is subsequently introduced into T cells via lentivirus or retrovirus transduction, followed by *in vitro* amplification and re-infusion into the patient [138]. The field of CAR-T cell therapy is rapidly advancing and continuously integrating with emerging technologies, such as CRISPR-based CAR-T cell therapy [139] and tLNP-mediated CAR-mRNA delivery for direct *in vivo* generation of CAR-T cells [140]. Most CARs primarily target proteins and glycosaminoglycans located on the cell surface (Table 2). However, recent research has demonstrated that CAR engineering can also effectively target intracellular peptide p16, which is delivered by MHC. p16 is a protein associated with cellular senescence and tumorigenesis. The engineered single-chain Fabs CARs have been designed to specifically identify and target senescent cells expressing p16 [141]. In addition, variable fragments of T cell receptor (TCR)-like antibodies in CAR-T cells recognize intracellular antigen-derived peptide/major histocompatibility complex (pMHC). This enables CAR-T cells to overcome cell surface barriers [142], presenting promising prospects for related therapies. The integration of these novel technologies broadens the application scope of CAR-T cell therapy and confers substantial benefits upon patients.

#### 3.3.1.2 CAR-T cell therapy in aging

**Figure 3.**
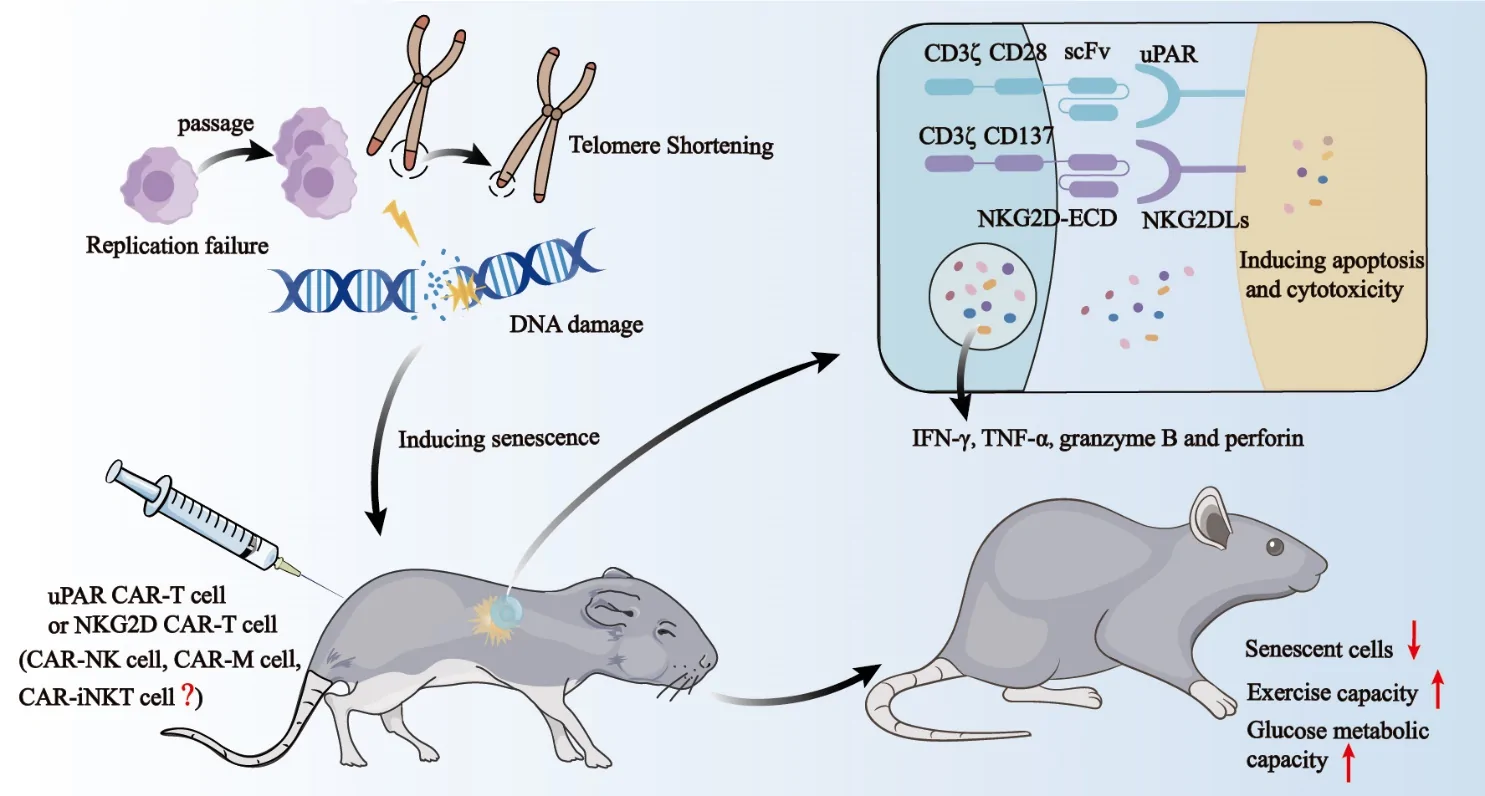
**The selective eradication of senescent cells using CAR-T cells**. The senescence state is triggered by internal or external stress, and the targeted elimination of senescent cells using uPAR CAR-T cells or NKG2D CAR-T cells enhances glucose tolerance and exercise capacity in aging models.

Adoptive therapy with antigen-specific CAR-T cells has the potential to mitigate aging and age-related diseases (Fig. 3). The previous CAR-T anti-aging study used m.uPAR-h.28z CAR-T cells, which contained the human CD28 co-stimulatory region and the CD3ζ signaling domain (h.28z). These cells were applied to senescent cells and showed excellent targeting ability and significant therapeutic effects. Experiments conducted at different dosages found that the appropriate use of m.uPAR-h.28z CAR-T cell treatment could clear senescent cells without causing severe cytokine release syndrome (CRS)-like symptoms. Finally, the regulatory effect of m.uPAR-h.28z CAR-T cells on cellular senescence was validated in a non-alcoholic steatohepatitis (NASH) model, effectively reversing liver fibrosis [143]. More excitingly, uPAR CAR-T cells also ameliorate age-related metabolic disorders and physical dysfunction and can maintain durability and preventive effects through activation of memory and effector CD8^+^ T cells [144].

Due to the expression of natural killer group 2 member D ligand (NKG2DL) by senescent cells, they can evade endogenous NK cell-mediated innate immunity [106]. Thus, CAR-T cells targeting NKG2DL were designed and successfully validated for clearance efficacy in a naturally aging mouse model and a closely related human macaque aging model. Importantly, hNKG2D-CAR T cells do not cause serious side effects in non-human primates [145]. The NKG2DL receptor is a promising target for CAR-T and CAR-NK cell therapies. Several CAR-T and CAR-NK cell therapies targeting NKG2DL have already entered clinical trials. Although NKG2D-CAR-T cells have shown good safety in clinical trials for cancer [146], it is necessary to conduct comprehensive clinical studies on their potential adverse reactions in the elderly population. For example, when using NKG2D-CAR-T cells for treatment in the presence of inflammation, bacterial or viral infections, the release of cytokines may exacerbate the infection and lead to a series of problems [145].

#### 3.3.1.3 Limitations of CAR-T cell therapy

At present, the biggest controversy in CAR-T cell therapy is its adverse reactions. The most prevalent manifestation is CRS, which presents as symptoms resembling influenza, capillary leakage, severe hypotension, shock, and multiple organ failure [147, 148]. CRS is also an important factor causing cardiovascular adverse events in CAR-T cell therapy [149, 150]. IL-1 and IL-6 from macrophage and monocyte-derived phagocyte lineages are important drivers of CRS-related toxicity [148]. The utilization of IL-6 receptor antagonists, such as tocilizumab, can significantly ameliorate CRS related toxicity [151]. Immune effector cell-associated neurotoxicity syndrome (ICANS) is also a prevalent and potentially life-threatening adverse reaction to CAR-T cell therapy [152]. The typical presentation of ICANS includes impaired consciousness and language dysfunction, while severe cases may progress to coma and seizures [153]. Both adverse reactions involve the interleukin family and can be managed hierarchically [154]. In addition, CAR-T cell therapy cannot be sustained for a long time [143], the reappearance of senescent cells has been observed approximately 6 months after NKG2D-CAR-T cell therapy [145]. The issue can be addressed by enhancing the persistence of CAR-T cells post-infusion through the selection of appropriate T cell sources, optimization of *in vitro* culture conditions and gene manipulation [155, 156]. These approaches offer valuable insights for potential applications in CAR-T-based anti-aging therapies. We consider the development strategies of dual-targeting CD19/CD20 [157], CD19/CD22 [158], and BCMA/CD38 CAR-T cells [159] in preclinical models and clinical trials of hematologic malignancies and multiple myeloma. The use of two different types of CAR-T cells with different antigen-binding specificities to target senescent cells with a high degree of heterogeneity may be an inspiration. The precise eradication of senescent cells through the design of dual-targeted CAR-T cells necessitates further investigation. Furthermore, the lack of clinical studies on CAR-T cell aging treatment to substantiate its safety and efficacy necessitates subsequent follow-up and reporting.

### 3.3.2 CAR-NK cells

After the successful application of CAR-T cells in the treatment of hematological malignancies, researchers' interest in CAR-NK cells has been piqued. CAR-NK cell therapy offers advantages such as enhanced safety and high feasibility for "off-the-shelf" production [160]. However, there is a lack of relevant research on CAR-NK therapy in anti-aging.

The targeting capacity of NK cells encompasses a wide range of diseases and has been extensively investigated in the context of cancer, infectious diseases, and autoimmune disorders. The utilization of adoptive transfer NK cells for the removal of senescent cells has demonstrated significant advancements and exhibited notable therapeutic efficacy in recent years. The study conducted by Bai et al. demonstrated a significant reduction in aging CD3^+^ T cells in peripheral blood through the adoptive transfer of NK cells and evaluated Acein-enhanced cytotoxicity of peripheral blood NK cells [161]. The sources of adoptively transferred NK cells include peripheral blood, cord blood, NK-92 cells, KHYG-1 cells, and iPSCs [162]. Engineered NK cells can also enhance immune system surveillance. By expressing CARs on NK cells, these cells can target senescent cells more accurately. Additionally, NK cells in the body can effectively identify and eliminate senescent cells through the NKG2D-NKG2DLs signaling pathway. Notably, the introduction of NKG2D-CAR-NKAE cells has demonstrated remarkable efficacy in treating multiple myeloma [163]. Therefore, NKG2DL as a target for anti-aging cell therapy is adapted to CAR-NK cell therapy to some extent, supporting the engineering transformation of NK cells expressing NKG2D-CAR. However, further follow-up studies are needed. In the field of cancer, compared to CAR-T cell therapy, CAR-NK cells do not elicit CRS and exhibit a shorter duration within the body than CAR-T cells, reducing the likelihood of inducing graft-versus-host disease and ensuring enhanced safety [164, 165].

In summary, CAR-NK cells, as a new type of cellular immunotherapy, represent a promising anti-aging approach. It is hoped that CAR-NK cell therapy can achieve breakthroughs in the field of anti-aging.

### 3.3.3 CAR-M cells

CAR-M cells are genetically engineered macrophage that expresses chimeric antigen receptors on their cell surface [166]. Recently, it has been reported that activation of the IL-4-STAT6 pathway to promote DNA repair effectively protects macrophages from aging and partly improves the healthy lifespan of aging mice [167]. In type 1 diabetes, β cells are initially induced to undergo premature aging, followed by the recruitment of macrophages to the islets for phagocytosis and elimination of senescent β cells. This process effectively prevents their functional decline and avoids terminal aging [168]. However, as individuals age, the selectivity and efficiency of macrophages tend to diminish. The utilization of CARs for redirecting macrophages towards recognizing specific antigens on cancer cells has been successfully developed for cancer treatment [169]. Currently, NCT04660929 and NCT05164666, registered on clinicaltrials.gov, are in Phase I clinical studies. CAR-M cells, similar to CAR-NK cells, are readily accessible and widely available. In addition to their phagocytic capabilities, CAR-M cells also enhance antigen presentation ability and activate the cytotoxicity of T cells [170]. The potential of CAR-M cells in anti-aging treatment lies primarily in their ability to selectively eliminate senescent cells. They can inhibit the aging process and delay the rate of aging. CAR-M cells have the potential to enhance the internal environment by effectively eliminating these senescent cells. This helps to maintain overall health. Macrophages possess the ability to produce inflammatory cytokines such as TNF-α, IL-6, IL-12, and IL-1β. That plays a crucial role in regulating immune responses and generating reactive oxygen species for efficient pathogen elimination [171, 172]. CAR-M cells may optimize immune responses to combat aging-related diseases. Moreover, CAR-M cells have less circulation time in the body and less toxicity to normal cells. Therefore, CAR-M cells for anti-aging will be a promising option in the future.

### 3.3.4 CAR-iNKTs

CAR-iNKT cells are natural killer T cells that carry a specific CAR [122]. It is known that α-GalCer can bind to CD1d to activate iNKT cells [116]. CD1d is an HLA class I-like molecule expressed in monocytes, macrophage dendritic cells, B cells, and thymocytes for glycolipid presentation [173]. However, the sufficiency of CD1d for iNKT cells to recognize senescent cells remains uncertain. Therefore, "navigation" CARs are introduced into iNKT cells to enhance their precise and rapid targeting of senescent cells. The migratory capacity and memory effect phenotype exhibited by iNKT cells enable them to regulate immune responses in extralymphatic tissues [174]. CAR-iNKT cells can recognize and attack tumor cells with corresponding antigens. Studies have shown that BCMA CAR-iNKT cells were amplified and persistent in tumor transplanted mice treated with rhIL-7-hyFc, significantly reducing tumor burden and prolonging survival [175]. Although iNKT cells are a rare subset of lymphocytes, they possess robust *in vitro* proliferative capacity [176, 177]. This is conducive to the development of CAR-iNKT cells expressing corresponding ligands to combat aging-related diseases. The potential and practical effects of CAR-iNKT cells in anti-aging therapy need to be further validated and explored through more preclinical and clinical studies.

## 4. Summary and prospects

Throughout the process of human aging, there are distinctive features including genomic instability, telomere attrition, epigenetic regulations, protein homeostasis loss, nutritional sensing disorders, mitochondrial dysfunction, macroautophagy dysfunction, cellular senescence, stem cell depletion, intercellular communication changes, chronic inflammation and gut microbial dysbiosis. We discuss a novel and challenging immune clearance strategy to address the issue of cellular senescence. This strategy is based on different antigen expressions, stimulating the immune system to recognize and clear senescent cells. In addition to mobilizing immune cells, the mainstream approach to cancer treatment-immunotherapy strategies that inhibit immune checkpoints can also be adjusted. This may offer a new way to intervene in aging. In recent years, CAR-T cell therapy has been successfully applied to a diverse range of diseases and has yielded remarkable outcomes, encompassing cancer, autoimmune disorders, inflammation, cardiovascular ailments, and fungal infections. However, due to issues such as potential toxicity and high cost associated with CAR-T cell therapy, there is a need to develop safer methods of cell programming in addition to integrating emerging technologies, such as CAR-NK and CAR-M cells. A major hurdle in CAR-related technologies is the absence of selective biomarkers for identifying senescent cells. By identifying specific targets for the precise or effective targeting of senescent cells, the installation of "navigational" CARs on immune cells can enhance specificity and effectiveness. Based on the success of double-target CAR-T cells in tumor treatment, we hope to apply dual-target CAR-T cells to anti-aging therapy aimed at removing senescent cells. This approach can compensate for the limited durability of single-target CAR-T cells. The combination of checkpoint blockade antibodies with CAR-T cells immunotherapy demonstrates superior clinical efficacy in cancer treatment compared to monotherapy [138]. This provides a new method for anti-aging treatment. Finally, we need to continuously explore new immunotherapy methods to eliminate senescent cells that are harmful to health and improve the quality of life.
